# Sex differences in cardiac transcriptomic response to neonatal sleep apnea

**DOI:** 10.14814/phy2.16110

**Published:** 2024-07-09

**Authors:** Emily C. Cheung, Anna Nilsson, Ian Venter, Grant Kowalik, Caitlin Ribeiro, Jeannette Rodriguez, Kiralee Kuraoka, Rebekah Russo, Joan B. Escobar, Bridget R. Alber, David Mendelowitz, Matthew W. Kay, Kathryn J. Schunke

**Affiliations:** ^1^ Department of Biomedical Engineering The George Washington University Washington District of Columbia USA; ^2^ Department of Pharmacology and Physiology The George Washington University Washington District of Columbia USA; ^3^ Department of Anatomy, Biochemistry & Physiology University of Hawaii Honolulu Hawaii USA

**Keywords:** autonomic imbalance, cardiac remodeling, cardiac miRNA expression, CIH, chronic intermittent hypoxia, transcription

## Abstract

Pediatric obstructive sleep apnea poses a significant health risk, with potential long‐term consequences on cardiovascular health. This study explores the dichotomous nature of neonatal cardiac response to chronic intermittent hypoxia (CIH) between males and females, aiming to fill a critical knowledge gap in the understanding of sex‐specific cardiovascular consequences of sleep apnea in early life. Neonates were exposed to CIH until p28 and underwent comprehensive in vivo physiological assessments, including whole‐body plethysmography, treadmill stress‐tests, and echocardiography. Results indicated that male CIH rats weighed 13.7% less than age‐matched control males (*p* = 0.0365), while females exhibited a mild yet significant increased respiratory drive during sleep (93.94 ± 0.84 vs. 95.31 ± 0.81;*p* = 0.02). Transcriptomic analysis of left ventricular tissue revealed a substantial sex‐based difference in the cardiac response to CIH, with males demonstrating a more pronounced alteration in gene expression compared to females (5986 vs. 3174 genes). The dysregulated miRNAs in males target metabolic genes, potentially predisposing the heart to altered metabolism and substrate utilization. Furthermore, CIH in males was associated with thinner left ventricular walls and dysregulation of genes involved in the cardiac action potential, possibly predisposing males to CIH‐related arrhythmia. These findings emphasize the importance of considering sex‐specific responses in understanding the cardiovascular implications of pediatric sleep apnea.

## 1 INTRODUCTION

Alterations to the supply of oxygen during early life pose a profound stress to physiological processes during critical periods of development, with potentially deleterious outcomes that can be long‐lasting. Obstructive sleep apnea (OSA) is a disorder characterized by the episodes of complete and partial upper airway obstruction resulting in intermittent hypoxia and sleep fragmentation. It occurs in approximately 1% to nearly 11% of all children and adolescents (Di Carlo et al., 2020; Kaditis et al., 2016; Marcus et al., 2012). Infants are particularly vulnerable to OSA due to multiple predisposing factors intrinsic to early life, including their small upper airway structure, immature pulmonary mechanics and ventilatory control, high arousal threshold, and a REM‐predominant sleep state. Untreated pediatric OSA often leads to developmental deficits, such as cognitive impairments, behavior disorders, metabolic disorders, and cardiovascular morbidities. Cardiovascular manifestations can range from overt hypertension, autonomic disruption, increased oxidative stress, systemic inflammation, endothelial dysfunction, reduced nocturnal baroreflex gain and subclinical abnormalities in cardiac structure and function (Aljadeff et al., 1997; Kaditis et al., 2010; Kheirandish‐Gozal et al., 2010).

Diagnosis of OSA is based on a combination of clinical and polysomnographic criteria; however because snoring or other less universal OSA symptoms are often used to identify subjects eligible for pediatric polysomnography, underestimation of OSA prevalence may occur, especially if clinical signs are absent or there is parental failure to recognize symptoms of OSA. Adenotonsillectomy (T&A) is typically the first line of treatment of upper airway obstruction, however significant residual OSA following T&A has become a growing concern (Bhattacharjee et al., 2010; Boudewyns et al., 2017; Gozal et al., 2020; Manickam et al., 2016). For children who are not candidates for T&A, or for whom T&A has failed, CPAP therapy is used and has been shown to be effective although adherence to recommended use, especially in younger children, remains a major barrier. Additionally, this management strategy fails to sufficiently address OSA treatment in the early postnatal population, whom often have more severe sleep apnea than older children and for whom T&A is rarely performed (Don et al., 2009; Katz et al., 2012).

Multiple recent studies have demonstrated that even with resolution of OSA at an early age, maladaptive remodeling in response to restricted oxygen during development imposes cardiovascular risk factors that have long‐term implications, well into adulthood. For example, children who had OSA have a higher prevalence of adult hypertension, obesity, autonomic dysfunction, and metabolic syndromes than adults without history of childhood OSA (Smith & Amin, 2019). Thus, early pathophysiologic changes may lead to a lifetime burden of cardiovascular disease and early mortality.

In adults there is a distinct sex bias in the prevalence ([AHI] ≥5; 34% men, 17% women (Yeghiazarians et al., 2021)), severity ([AHI] ≥15; 13% men, 6% women (Peppard et al., 2013)), presentation, and cardiovascular consequences of OSA (Geovanini et al., 2018). These statistics may; however, be partially influenced by the fact that studies in women and female animal models are underrepresented in cardiac research, despite cardiovascular disease being the primary killer of women in the United States. In the limited reports that do include both sexes, significant sexual dimorphisms have been demonstrated in development, presentation, and outcome of cardiovascular disease, with many studies reporting discrepant results. Recent studies that have included larger female subject sample sizes suggest that females are often underdiagnosed and may have similar or worse OSA associated cardiovascular outcomes than men (Bonsignore et al., 2019; Bouloukaki et al., 2021; Kendzerska et al., 2020). This limited understanding of the mechanisms underlying the sexual dimorphism that may initiate and sustain sex‐biased cardiovascular risk to sleep apnea, is a barrier to cardiovascular disease prevention and treatment. Importantly, there have been few studies examining the influence of sex on cardiovascular outcomes in neonates with OSA. Therefore, the aim of this study was to determine the effect of sex on the cardiac response to chronic intermittent hypoxia (CIH), as a model of sleep apnea, in postnatal rats.

## 2 METHODS

### 2.1 Chronic intermittent hypoxia exposure

**FIGURE 1:**
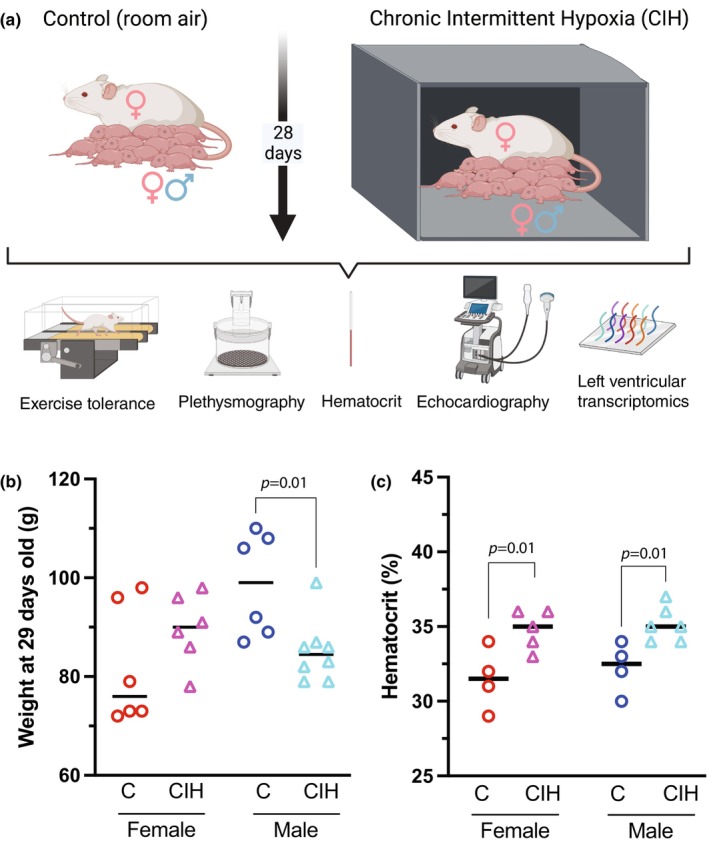
(a) Protocol schematic depicting dams with female and male pups (P1) exposed to either room air (control) or chronic intermittent hypoxia (CIH) for 28 days. Outcome measures consisted of in vivo treadmill exercise tolerance tests, whole‐body plethysmography, echocardiography, and ex vivo hematocrit and left ventricular transcriptomics. Created with BioRender.com. (b) Body weight of pups at P29 was significantly lower in males exposed to CIH than controls (*n* = 6–8 animals per group, one‐way ANOVA). (c) Hematocrit at P29 was significantly greater for female and male pups exposed to CIH than their sex and age matched controls (*n* = 4–6 animals per group, one‐way ANOVA).

Sprague–Dawley dams and their male and female pups, from age P1–P28, were exposed to intermittent cycles of low oxygen (CIH) during their sleep period using an approach similar to that of our previous studies (Figure 1a) (Jameson et al., 2016; Rodriguez et al., 2023). Animals, inside their cages with normal bedding and unrestricted access to food (LabDiet 5V75; Richmond, IN) and water, were placed inside sealed commercial chambers with computer‐controlled atmospheric gas regulation (Biospherix) for 8 h each day (10:30 a.m. to 6:30 p.m.). Sensors in the chambers monitored the oxygen concentration. Room air and nitrogen were cycled within the chamber until each defined oxygen set point was reached. The CIH protocol cycled between room air (21% O_2_, 79% N_2_) and hypoxia (6% O_2_, 94% N_2_) 10 times per hour in 4 phases: (1) room air to hypoxia in 90 s, (2) maintained hypoxia for 120 s, (3) hypoxia to room air in 90 s, and (4) maintained room air for 90 s. Animals were returned to the vivarium each day at the end of the CIH protocol. This CIH protocol closely imitates the hypoxic events that occur during obstructive events in OSA but does not include the hypercapnia that often also occurs with apneas and hypopneas. Pups were weaned at P21 and continued CIH until P28. Control pups and dams (breathing room air) were kept in the animal facility and weaned at P21. All animals were sacrificed by exsanguination and heart removal under isoflurane anesthesia on day P29. The four experimental groups were (1) female control (*n* = 6), (2) female CIH (*n* = 6), (3) male control (*n* = 6), and (4) male CIH (*n* = 8). Each experimental group was composed of pups from three different dams.

### 2.2 In‐vivo physiological measurements

At P24‐P28, both the CIH (*n* = 14, 8M, 6F) and normoxic control animals (*n* = 12, 6M, 6F) received whole body plethysmography, treadmill test, and echocardiogram **(**Figure 1a **)**. Breathing was monitored through vivoFlow whole‐body plethysmography (SCIREQ Scientific Respiratory Equipment Inc., Montréal, Canada). The rats remained conscious throughout the entire recording. Rats were given 1 h of acclimation followed by a 1.5 h recording between the hours of 9 a.m. and 12 p.m..

The treadmill protocol measured peak effort capacity, determined by the rats' ability to run on the enclosed treadmill until the point of exhaustion. Each rat received a 5‐min acclimation period to the treadmill followed by a starting speed of 8 cm/sec, which increased in increments of 5 cm/s every 3 min until exhaustion. To increase the rat's motivation, bursts of compressed air were sprayed into the backend of the treadmill. Indications of end‐point exhaustion included cessation of response to compressed air and the usage of treadmill walls for support.

Echocardiographic measurements were taken 2 days before sacrifice on lightly anesthetized (1.5% isoflurane inhalation) rats using the Visualsonics small‐animal system (Vevo 3100 Imaging System, FUJIFILM Visual Sonics Inc, Toronto, Ontario, Canada), as described previously (Dyavanapalli et al., 2020; Schunke et al., 2023). LV outflow tract diameter was measured from a 2D‐parasternal LV long axis view. M‐mode images from a parasternal LV short axis view at the level of the papillary muscles were used to measure LV structural variables and to calculate fractional shortening (FS). Inflow velocities through the mitral valves were measured using Pulsed‐Wave Doppler imaging in an apical four chamber view to assess early diastolic mitral inflow (E wave), late diastolic mitral inflow (A wave), and early to late mitral inflow (E/A) ratio. Functional variables, including ascending aorta blood flow velocity, HR, and cardiac output (CO) were measured from continuous wave Doppler signals.

### 2.3 Tissue and blood collection

Rats were anesthetized with 5% isoflurane inhalation. After voluntary pain reflexes ceased, blood was collected in K3 EDTA tubes from the inferior vena cava prior to sacrificing and used to measure hematocrit (9500 RPM; 6 min). The heart was rapidly excised and rinsed in PBS, followed by separation of the left and right ventricle that were subsequently flash frozen in liquid nitrogen.

### 2.4 Plethysmography analysis

Plethysmography data files generated by the SCIREQ vivoFlow analysis software were analyzed in MATLAB to extract values of frequency of breathing, tidal volume, peak inspiratory flow, peak expiratory flow, and minute ventilation. Periods of wakefulness and rest were differentiated by defining rest as three or more consecutive breaths less than 100 bpm. Apnea analysis was subsequently performed on the raw respiratory waveform using Labchart software (ADInstruments, Colorado Spring, CO). Apneas were defined as pauses in breaths lasting longer than three times an average breath, with the threshold set to 1.25 s.

### 2.5 Transcriptomics of LV tissue

Flash frozen LV tissue was cryo‐pulverized and total RNA isolated, according to the manufacturer's protocol, using miRNeasy Tissue/Cells Advanced Kit (Qiagen, Germantown, MD, #217684). Isolated RNA was quantified using a Qubit Fluorometer (Invitrogen ThermoFisher Scientific) and assessed for quality using an Agilent 2100 Bioanalyzer (Agilent Technologies). An Affymetrix Clariom D Array (ThermoFisher, Waltham, MA, #902632; *n* = 4 hearts per group) and GeneChip miRNA 4.0 Assay (ThermoFisher, #902413; *n* = 4 hearts per group) were used for transcriptome and miRNA profiling, respectively. Normalization of the transcriptomic data was performed by SST‐RMA summarization to generate gene level expression signals using Transcriptome Analysis Console (TAC 4.0, Applied Biosystems) software after the evaluation of quality control metrics. Differentially expressed genes (DEGs) were identified using TAC with filtering criteria of 1.4 < fold change < −1.4 and *p* < 0.05 for the Clariom D Array and 2 < fold change < −2 and *p* < 0.05 for GeneChip miRNA 4.0 data. Prediction of gene targets for differentially expressed miRNA were filtered using “high” and “moderate” prediction of “miRNA Confidence” metric. Ingenuity Pathway Analysis (IPA) software (Qiagen) was used to analyze biological pathways of differentially expressed genes and miRNA regulated genes.

### 2.6 Semiquantitative real‐time PCR

Real‐time semi‐qPCR was used to confirm the expression fold changes of key genes measured using the Affymetrix Clariom D Assay. cDNA was generated from individual hearts (Quanta Biosciences, Beverly, MA, #95047–100; *n* = 4 per group) and oligonucleotide primers (Integrated DNA Technologies, Coralville, IA) were designed to cross an intron (Table S1; Figure S1). qPCR was performed on cDNA representing 5 ng of total RNA. PCR was run in triplicate with PowerUp SYBR Green (ThermoFisher, #A25742). Gapdh which was unregulated by any group on microarray analyses, was used as an internal control.

### 2.7 Statistical analyses

Data were analyzed using GraphPad Prism and presented as mean ± standard deviation. The Shapiro–Wilk test for normality, followed by either a Student's *t*‐test or the Mann–Whitney nonparametric test, was used when appropriate. A one‐ or two‐way ANOVA with Šídák's test for multiple comparisons was used to compare differences between groups, when appropriate. To compare differences in mRNA expression between groups, multiple unpaired Student's *t*‐tests were performed followed by the two‐stage linear step‐up procedure of Benjamini, Krieger, and Yekutieli test for multiple comparisons. Results were considered statistically significant if *p* < 0.05.

## 3 RESULTS

### 3.1 CIH males weigh less

We compared the effects of postnatal (P1‐P28) CIH on body weight in female and male rats. As expected, control females weighed significantly less than age matched control males. Interestingly, male CIH rats weighed 13.7% less than (*p* = 0.0365) age‐matched control males, however CIH did not significantly affect female weight. (Figure 1b). Both female and male CIH rats had similarly elevated hematocrit compared to controls (31.5 ± 2 vs. 34.8 ± 1.3 and 32.25 ± 1.7 vs. 35.2 ± 1.2, for females and males, control vs. CIH, respectively), supporting similar levels of sustained systemic hypoxia exposure between males and females (Figure 1c).

### 3.2 CIH does not affect treadmill exercise performance

**FIGURE 2:**
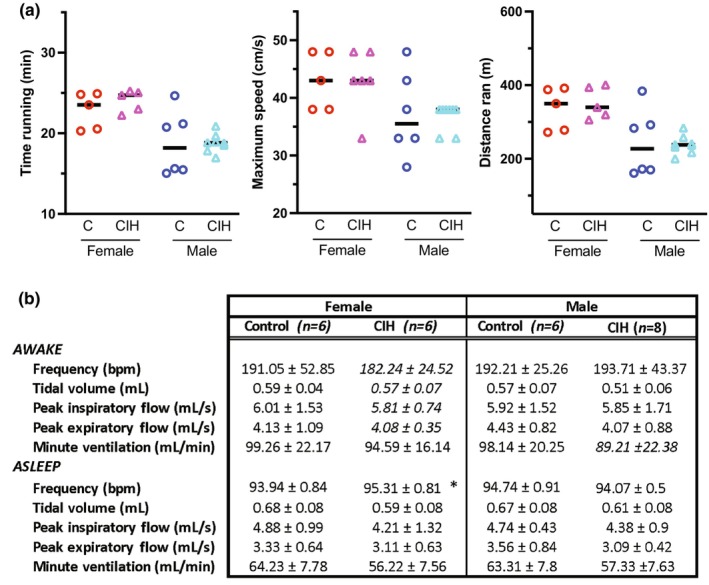
(a) Treadmill exercise tolerance tests performed at P24‐P28 illustrates that both female and male rats exposed to chronic intermittent hypoxia (CIH) perform no differently than their sex and age matched controls (*n* = 6–8; one‐way ANOVA). (b) Whole‐body plethysmography performed at P24‐P28 identify that females exposed to CIH have increased breathing frequency during sleep than their age matched controls (*n* = 6–8 animals per group, one‐way ANOVA; *p = 0.02).

Maximal exercise capacity tests are often used as an indicator of cardiorespiratory capacity (Rodrigues et al., 2007). Using a rodent treadmill peak effort protocol, we found that 28 days of neonatal CIH exposure had no significant effect on running duration, maximum speed achieved, nor distance ran, in females or males (Figure 2a).

### 3.3 CIH increases respiratory drive during sleep in females

After neonates were exposed to 26 days of CIH (P1‐P26), we used normoxic whole body plethysmography to assess changes in baseline respiration. We assessed breathing frequency (bpm), tidal volume (mL), peak inspiratory flow (mL/s), peak expiratory flow (mL/s), and minute ventilation (mL/min) during both wake and sleep phases. During the sleep phase, CIH females breathing frequency was mildly but significantly greater than control females, while male frequency did not change (Figure 2b).

### 3.4 Males have an exaggerated cardiac transcriptomic response to CIH

**FIGURE 3:**
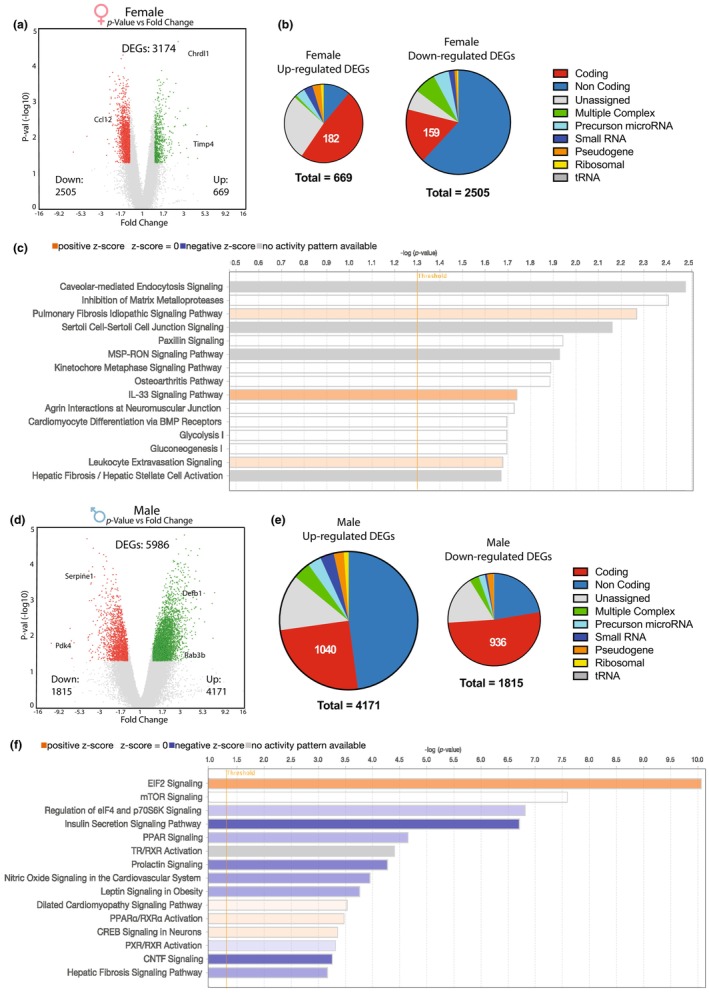
Transcriptome analysis of LV myocardium differentially expressed genes (DEGs). (a, d) Volcano plots of DEGs (1.4 < FC < −1.4, *p* < 0.05; *n* = 4 per group) between chronic intermittent hypoxia (CIH) and control females (a) and males (d). (b, e) Up‐ and down‐regulation of differentially expressed nucleotide sequence categories between CIH and control females (b) and males (e) show that females primarily reduce transcription while males primarily enhance transcription in response to CIH. (c, f) Top 15 biological canonical pathways represented by the DEGs in (b, e). The bar chart depicts −log significance (upper x‐axis) of the differential pathway regulation with the threshold for significance (vertical orange line) set at −log 1.3 (*p* < 0.05). Bar color represents either upregulation (orange) or downregulation (blue) of the pathway with more intense color gradation corresponding to a higher percentage of DEGs enriched within a given biological pathway.

To understand the underlying biological processes affected by CIH exposure in neonates, we analyzed the gene expression profile changes in left ventricular tissue. Hearts of male rats had a greater number of genes that significantly changed in response to CIH (DEGs; differentially expressed genes) than females (5986 vs. 3174 genes) (Figure 3a,d and Table S2). Females responded to CIH primarily by down‐regulating (2505 genes) versus up‐regulating gene expression (669 genes) (Figure 3b). Conversely, males primarily up‐regulated genes (4171 up vs. 1815 down) (Figure 3e). In males, 33% of the DEGs were coding genes versus only 11% in females; however, females primarily downregulated coding genes. Interestingly, the 3174 female DEGs were not strongly enriched in particular biological pathways (Figure 3c). Of the top 15 female canonical pathways only three had activity patterns (z‐score), which were all positive, suggesting activation of the pathway. The pathway with the strongest activation score was IL‐33 Signaling (5/186 genes), and the second was Leukocyte Extravasation Signaling. Activation of these pathways suggest inflammation of the heart, and are associated with hypertension and microvascular complications (Ghali et al., 2018; Zychowski et al., 2016).

Conversely, male DEGs were highly enriched in many biological pathways and primarily had negative z‐score activity patterns (Figure 3f). Pathways related to growth and nutrient utilization (Regulation of eIF4 and p70S6K Signaling, Insulin Secretion Signaling, PPAR Signaling, Leptin Signaling in Obesity) were all inhibited, suggesting cardiac metabolic dysregulation. Likewise, reduced Prolactin Signaling and Nitric Oxide Signaling in the Cardiovascular System pathway activity is associated with autonomic dysfunction, hypertension, and cardiovascular disease (Hare & Colucci, 1995; Papazoglou & Leite, 2023). The EIF2 Signaling pathway, which promotes protein synthesis, was the most significantly enriched pathway in males and had a positive z‐score.

### 3.5 Dysregulated miRNAs target metabolic genes in CIH males

**FIGURE 4:**
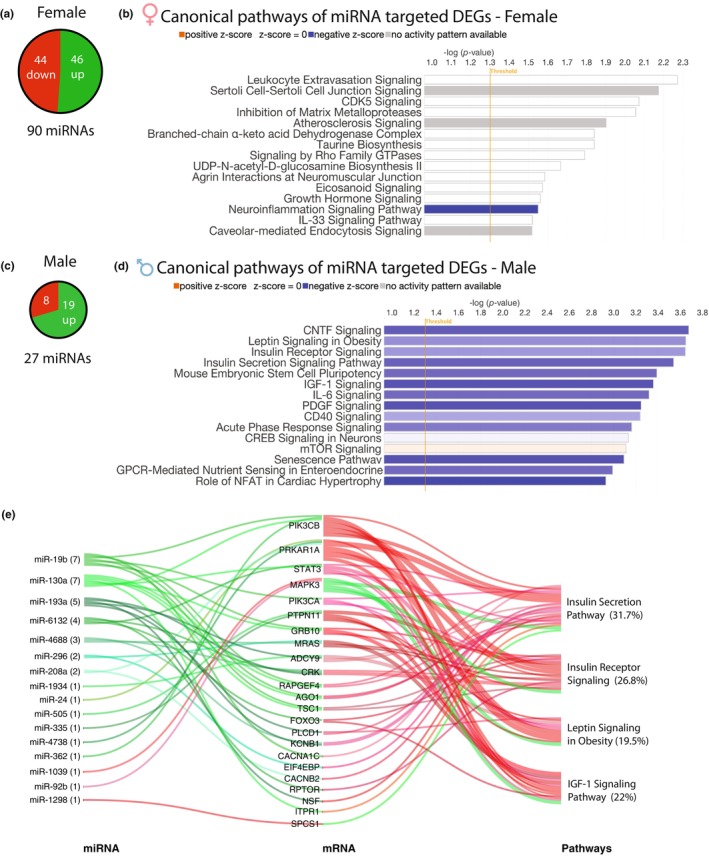
miRNA analysis of LV myocardium. (a, c) Total number and up‐ or down‐regulation of differentially expressed miRNAs (2 < FC < −2, *p* < 0.05; *n* = 4 per group) in female (a) and male (c) neonatal animals exposed to chronic intermittent hypoxia (CIH) compared to age and sex matched controls. (b, d) Top 15 biological canonical pathways represented by the differentially expressed genes (DEGs) that are targets of miRNAs in (a, c). The bar chart depicts −log significance (upper x‐axis) of the differential pathway regulation with the threshold for significance (vertical orange line) set at −log 1.3 (*p* < 0.05). Bar color represents either upregulation (orange) or downregulation (blue) of the pathway with more intense color gradation corresponding to a higher percentage of DEGs enriched within a given biological pathway. (e) Sankey chart of miRNAs dysregulated in male neonatal CIH exposure, the DEGs (mRNA) within the dataset they are predicted to regulate and the metabolic pathways those DEGs are enriched in. Number to the right of the miRNA represents the number of DEGs within the dataset that miRNA targets. Green line leaving miRNA or mRNA illustrates that gene is upregulated; red line leaving miRNA or mRNA illustrates that gene is downregulated within the dataset.

Since small RNA and precursor miRNA made up 5.4% of DEGs in male and 6.8% of female DEGs, we used a miRNA GeneChip 4.0 to determine which mature miRNAs were dysregulated. Filtering of miRNAs in TAC software (2 < fold change < −2; *p* < 0.05) was performed, then IPA software was used to predict gene targets of the miRNAs. We found that although females have fewer DEGs than males, they have a greater number of differentially expressed miRNAs. Females had 90 dysregulated miRNAs with 51% upregulated (46 up and 44 down) while males had only 27 significant miRNAs with 70% of those upregulated (19 up and 8 down) (Figure 4a,c). miR‐374b was the only differentially regulated miRNA shared between females and males. We further examined if the predicted target genes for these miRNAs were dysregulated in the mRNA gene dataset. In both males and females, every differentially expressed miRNA was predicted to target at least one of the DEGs (Table S3). Female IPA pathway analysis of predicted miRNA regulated DEGs did not suggest enrichment of DEGs into any particularly pathways, with the exception of the Neuroinflammation Signaling pathway with a nominal 3 gene enrichment (Figure 4b). However, pathway analysis of predicted miRNA regulated male DEGs suggested multiple pathways related to metabolism (Leptin Signaling in Obesity, Insulin Secretion Signaling, Insulin Receptor Signaling, and IGF‐1 Signaling), and cell growth/proliferation and survival (CNTF signaling, PDGF signaling, mTOR signaling, GPCR‐mediated nutrient sensing in enteroendocrine cells) (Figure 4d). Importantly, all of these pathways (except for mTOR) had a negative z‐score, indicating downregulation of these processes in the heart due to CIH. The Sankey chart in Figure 4e illustrates that 16 of the 27 male miRNAs target 23 DEGs that are integral pathway components of the 4 downregulated metabolic pathways in Figure 4d. These data suggest that in males, miRNA dysregulation may drive altered expression of genes responsible for cardiac metabolism and survival, ultimately predisposing the heart to hindered metabolism and fuel usage. However, in females, miRNAs don't appear to target any particular pathway to inhibit or contribute to CIH cardiac pathogenesis/alterations.

### 3.6 miRNAs may contribute to thinner left ventricular wall in CIH males

**FIGURE 5:**
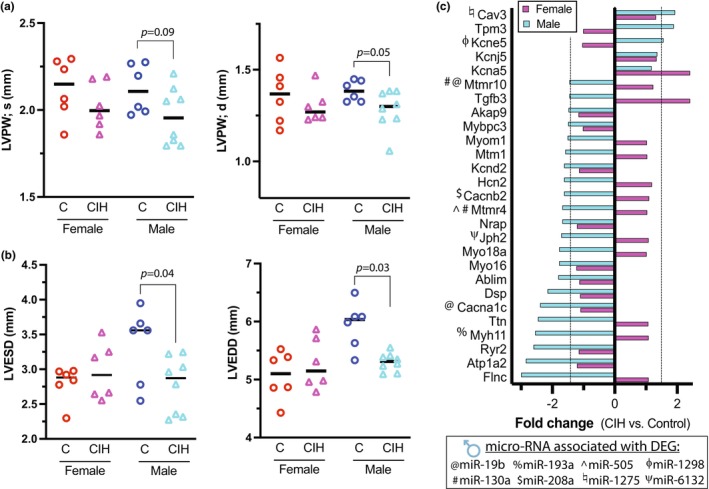
Cardiac functional and transcriptional response to neonatal chronic intermittent hypoxia (CIH). (a) Echocardiographic measures of left ventricular posterior wall thickness in systole (LVPW;s) or diastole (LVPW;d) (*n* = 6–8 animals per group, one‐way ANOVA). (b) Left ventricular chamber diameter in systole and diastole (LVESD, LVEDD) is smaller in males exposed to neonatal CIH (*n* = 6–8 animals per group, one‐way ANOVA). (c) Expression of key cardiac contractile, ion channel and communication genes in females and males exposed to neonatal CIH, compared to their respective age and sex matched controls. Dotted vertical line indicates the fold change cut‐off for significance (1.4 < FC < −1.4, *p* < 0.05; *n* = 4 per group). The table below lists male differentially expressed miRNAs (2 < FC < −2, *p* < 0.05; *n* = 3 per group) with a symbol corresponding to the differentially expressed genes (DEGs) it is predicted to regulate in the dataset.

Early cardiac functional or anatomic alterations due to OSA may predispose the heart to progressive disease, as arrhythmia, myocardial ischemia and heart failure are common comorbidities in OSA patients. Using echocardiogram measurements, we found cardiac systolic functional indices, such as fractional shortening, were not significantly affected by CIH in females (45.26 ± 3.27 control vs. 44.01 ± 3.27 CIH), or males (43.73 ± 5.2 control vs. 47.88 ± 6.55 CIH). Indexes of diastolic function such as E/A ratio were likewise not significantly different due to CIH in male or female animals. Left ventricular free wall diameter in CIH males trended thinner than control in systole (*p* = 0.09) and diastole (*p* = 0.05) (Figure 5a). However, left ventricular chamber diameter was significantly affected by CIH in males, but not females (Figure 5b). We examined sarcomeric gene expression to determine if differential gene expression contributed to thinning of the ventricular wall in males versus females. We found many sarcomeric related genes (Tpm, Mtmr10, Tgfb3, Mybbpc3, Myom1, Mtm1, Mtmr4, Nrap, Myo18a, Myo16, Ablim, Ttn, Myh11, and Flnc) were significantly down‐regulated in CIH males, but not females (Figure 5c). Interestingly, many of these sarcomeric genes are miRNA targets of male differentially expressed miRNAs in the dataset (miR‐130a, miR‐19b, miR‐505, and miR‐193a) which may be a mechanism responsible for reducing expression of the sarcomeric DEGs in males but not females.

### 3.7 miRNAs target differentially regulated cardiac action potential genes in CIH males

In adults, OSA is associated with ventricular arrhythmia and is an independent risk factor for atrial fibrillation (Yeghiazarians et al., 2021); however, incidence of arrhythmias in children with OSA is generally low. The mechanisms mediating adult arrhythmia are still uncertain, thus postnatal compensatory alterations in electrophysiology may give insight into adult manifestation. We found that many key cell communication (Cav3, Jph2, and Dsp) and cardiac actional potential modifying (Kcne5, Kcnj5, Akap9, Kcnd2, Hcn2, Cacnb2, Cacna1c, Ryr2, and Atp1a2) genes were dysregulated in CIH males compared to females (Figure 5c), where miR‐1275, miR‐1298, miR‐208a, miR‐6132, and miR‐19b were male CIH differentially expressed miRNAs that target the aforementioned cell communication and action potential DEGs. Kcna5 and Tgfb3 were the only genes in these categories dysregulated in female CIH animals, which were not targets of any differentially expressed miRNA in the female dataset (Figure 5c). These data suggest that neonatal males exposed to CIH may be more susceptible to electromechanical disruption than CIH females, and that miRNAs may be a regulatory mechanism in these changes.

## 4 DISCUSSION

This study demonstrates distinct sex dichotomy in the cardiovascular response to neonatal CIH, as a model of sleep apnea. Specifically, using transcriptomic profiling of the left ventricle, we identified early postnatal cardiac molecular characteristics of female and male hearts, which provide comparative insights into the early molecular changes in the myocardium associated with neonatal CIH. The importance of these observations stems from the knowledge that sex is an independent risk factor for future cardiovascular disease, and the origins of cardiovascular disease are firmly placed in childhood and are progressive during the lifecycle (Halvorsen et al., 2006; Schulz, 2010; Strong & McGill Jr., 1962; Sun et al., 2009). Early identification of individuals at increased risk of cardiovascular disease is fundamental to developing effective prevention and treatment.

### 4.1 Respiratory response

In the cardiovascular system, altered respiration rate and heart rate are the first compensatory responses to stimuli. In adult rodents, CIH induces alterations of the baseline central respiratory drive (Baker & Mitchell, 2000). These alterations are likely due to potentiation of peripheral chemoreceptors (Baum et al., 2018), and loss of serotonin and noradrenaline terminals in the hypoglossal nucleus of the brainstem (Sood et al., 2003). In female and male neonatal rats exposed to CIH, it has been noted that diaphragm development and function is not altered (McDonald et al., 2016), but that the carotid body is sensitized and augments the hypoxic ventilatory response (Peng et al., 2004). Another study found that the CIH‐induced sensitization of neonatal carotid bodies was not reversed after re‐exposure to normoxia as it was in animals exposed to CIH as adults, and further described that CIH was ineffective at inducing sensory long‐term facilitation (LTF) in neonatal carotid bodies (Pawar et al., 2008). Conversely, Julien et al. (2008) reported that the hypoxic ventilatory response was enhanced in only male rats exposed to neonatal CIH and ventilatory LTF was reduced in both sexes exposed to neonatal CIH. Our study shows that after 26 days (P1‐P26) of CIH, females significantly increased breathing frequency during sleep, while male breathing frequency did not change. These data indicate that female neonatal rats exposed to CIH, but not males, exhibit LTF. CIH‐induced LTF is dependent on serotonergic mechanisms (Bach & Mitchell, 1996), as LTF can be eliminated by 5‐HT_2_ receptor antagonism (Ling et al., 2001). Using immunohistology, Baum et al. (Baum et al., 2018) reported that adult females exposed to CIH exhibited greater brainstem neuroplastic dynamics and preserved serotonergic systems, resulting in better compensation to CIH stimulation than males.

We also found that CIH had no functional effects on the postnatal cardiorespiratory response to exercise stress. These data may indicate that compensatory mechanisms in response to CIH insult are effective postnatally and have not become maladaptive to the point of exhibiting a decline in cardiorespiratory function during exertion.

### 4.2 Metabolic response

Male postnatal CIH rats weighed significantly less than their age‐matched controls, yet female rat weight was not affected by neonatal CIH exposure. Although many initial reports of pediatric OSA described a disproportionate number of children with failure to thrive, this phenomenon is currently far less common since diagnosis and intervention have increased in OSA patients. Interestingly, even obese children with OSA will demonstrate accelerations in weight gain after treatment of OSA (Everett et al., 1987; Freezer et al., 1995). The proposed mechanisms for somatic growth alterations in OSA involve decreased levels of insulin‐like growth factor‐I, insulin‐like growth factor binding proteins, and possibly growth hormone since serum levels are decreased in children with OSA but improve following T&A or CPAP (Bar et al., 1999; Farmarzi et al., 2016; Nieminen et al., 2002). These data support the cardiac molecular signatures generated from transcriptomic data in this study. Downregulation of pathways in male CIH rats related to cell growth/proliferation and survival (CNTF signaling, PDGF signaling, mTOR signaling, GPCR‐mediated nutrient sensing in enteroendocrine cells) in the heart are likely in response to early systemic metabolic alterations. Furthermore, pediatric studies have shown that both mild and severe OSA are associated with metabolic disease and insulin resistance, independent of body weight (Bhushan et al., 2014; Isobe et al., 2019; Shamsuzzaman et al., 2014). Our data show significant differentially regulated gene enrichment in pathways related to insulin resistance and metabolic syndrome: Regulation of eIF4 and p70S6K Signaling, Insulin Secretion Signaling, PPAR Signaling, Leptin Signaling in Obesity. These data suggest that metabolic derangements occur in male neonatal CIH exposure, and may influence cardiac substrate utilization, ultimately predisposing the male heart to functional and/or structural changes. Furthermore, male differentially regulated miRNAs that target genes in these pathways suggest a sex‐biased epigenetic mechanism of regulation. Sexually dimorphic mRNA and miRNA expression networks targeting the cardiovascular response have also been reported in adult human and murine hearts (Tsuji et al., 2017).

### 4.3 Cardiac electrophysiology

OSA is associated with arrhythmia in at least 50% of adult patients (Somers et al., 2008), with atrial and ventricular tachyarrhythmias, bradyarrhythmias, prolongation of the QT interval, and sudden cardiac arrest (Gami et al., 2007; Shamsuzzaman et al., 2015). Incidence of arrhythmias in children with OSA is generally low and benign, although there are limited reports describing this relationship, especially in the younger pediatric population (Khositseth et al., 2013; Thomas et al., 2022). In a small cohort of children with OSA, P‐wave dispersion was present and more evident in severe versus mild OSA cases (Kraikriangsri et al., 2019). P‐wave dispersion is a predictor of atrial fibrillation and associated with early‐onset hypertension (Jagannatha et al., 2024). QT dispersion is also associated with an increased risk for ventricular arrhythmia and has been reported among children with more severe OSA (Khositseth et al., 2011). Recent reports suggest sex dichotomy in adult arrhythmia with lower burden and later onset of atrial arrhythmia in females (Lin et al., 2023) with OSA. These clinical data support our observations that multiple cardiac action potential genes and cell communication genes are dysregulated in males exposed to neonatal CIH, but not females. For example, prolonged QT interval is associated with delayed repolarization and is linked with mutations in multiple cardiac ion channel genes such as Cav3, Cacna1, Kcne1, Kcne2, and Kcnj2. Male, but not female, CIH rats have altered expression of Cav3, Cacna1c, Cacnb2, Kcne5, Kcnd2, and Hcn2 which may preferentially predispose postnatal males to develop arrhythmia. Ryr2, Atp1a2, and Cacna1c were also dysregulated in the male postnatal CIH animals, which may indicate altered calcium handling, a phenomenon previously reported in adult male hearts exposed to CIH (Yeung et al., 2007). Kcna5 and Tgfb3 were the only significant female DEGs associated with cardiomyocyte communication, both with increased expression.

### 4.4 Endothelial and autonomic mechanisms of hypertension

The molecular signature of female postnatal CIH rats suggests inflammation and endothelial dysfunction in the heart. Specifically, IL‐33 Signaling and Leukocyte Extravasation Signaling pathways were positively enriched, and activation of these pathways is associated with hypertension and microvascular complications (Ghali et al., 2018; Zychowski et al., 2016). Adult female patients with moderate OSA have been reported to have more severe endothelial dysfunction than male patients (Faulx et al., 2004); however, a recent report suggests that subclinical coronary atherosclerosis is equally associated with OSA in adult women and men (Medeiros et al., 2017).

Biological pathways that were downregulated in male CIH animals are also associated with hypertension and autonomic dysfunction (Bryan, 2022; Schultz, 2009): Nitric Oxide Signaling in the Cardiovascular System, and Prolactin Signaling. Interestingly, a recent longitudinal study found that childhood OSA was associated with adolescent hypertension in males but not females (Fernandez‐Mendoza et al., 2021). Moreover, the reduction in enhanced cardiac sympathetic nerve activity after CPAP therapy in OSA patients was more pronounced in male than female patients (Özlek et al., 2020). These conflicting data could implicate that the mechanism responsible for vascular perturbations and hypertension could be sex‐specific and originate early in life, with males more prone to nitric oxide and autonomic disturbances, and females more prone to inflammatory, atherosclerotic and microvascular perturbations.

### 4.5 Left ventricular geometry

Echocardiographic evidence for left ventricular hypertrophy, right ventricular hypertrophy, and decreased left ventricular function (often without clinical symptoms) have been reported in children with OSA (Amin et al., 2002, 2005). There is a trend toward increased prevalence of abnormal left ventricular geometry with increased severity of pediatric OSA, independent of sex (Amin et al., 2002). In the current study, the enlarged left ventricular chamber and trend toward thinner left ventricular wall in males may be indicative of an intermediate phenotype that could predispose males toward progressive cardiac remodeling and morbidity over time. These structural echocardiographic findings are further supported by transcriptional analysis of contractile genes which were altered primarily in male CIH animals. Hypertrophic and dilated cardiomyopathy causing mutations have been detected mostly in genes of sarcomere proteins such as Mybpc2, Myh7, Tnnt2, Tnnc1, Tnni3, Tpm1, Ttn, Actc1, Myl2, and Myl3 (van der Velden & Stienen, 2019). We found that multiple sarcomeric related genes (Tpm, Mtmr10, Tgfb3, Mybbpc3, Myom1, Mtm1, Mtmr4, Nrap, Myo18a, Myo16, Ablim, Ttn, Myh11, and Flnc) were significantly down‐regulated in CIH males, but not females. These data suggest that males exposed to neonatal CIH have a greater predisposition for structural and/or functional alterations compared to females. Interestingly, many of these sarcomeric genes are miRNA targets of male differentially expressed miRNAs in the dataset (miR‐130a, miR‐19b, miR‐505, and miR‐193a) which may also be a sex‐ dichotomous mechanism responsible for reducing expression of the sarcomeric DEGs in males but not females.

## 5 SUMMARY

This is the first study to describe sex‐dichotomy in the cardiac left ventricular transcriptomic responses to neonatal CIH, as a model of sleep apnea. Males exposed to CIH have significantly more DEGs than females, compared to their age and sex‐matched controls. Biological pathways enriched by the DEGs are robustly related to metabolism, growth, and autonomic dysfunction in males, while females have less significant enrichment in pathways related to inflammation and endothelial dysfunction. Left ventricular geometry is altered in males but not females, with corresponding differential regulation of contractile and electrophysiologic genes in males. Analysis of miRNA transcripts and target DEGs suggest that miRNAs may be a mechanism responsible for regulating DEGs associated with metabolic pathways and cardiac contraction in males, but not females. We further demonstrate that neonatal CIH induces respiratory LTF in females, and reduced weight gain in males. Overall, our findings demonstrate a relationship between early‐life exposure to CIH and changes in specific physiological outcomes, and provides insight into the molecular mechanisms associated with neonatal CIH that may contribute to cardiovascular morbidity.

### 5.1 Limitations

Although transcriptomic analysis is vital to gain insight into pathophysiologic mechanisms, differential mRNA expression does not always translate to differential protein expression or divergence in functional activity. Detection of post‐transcriptional and post‐translational modifications at the protein level are necessary to determine function of differentially expressed genes and their active state. Furthermore, because transcriptomic analysis was performed on heterogenous left ventricular tissue, this study cannot identify cell‐type specific responses that are invaluable to determine sex‐dichotomous pathophysiologic mechanisms, especially as they relate to the vasculature. Although IPA predictive software of miRNA gene targets is a powerful tool, it does not prove miRNA action causative for target gene differential expression in this study. CIH exposure has been associated with pulmonary and right ventricular structure/function alternations which were not assessed in this report. Observed changes in animal weight could be due to growth restriction, muscle mass or adiposity. Finally, although this study provides information about acute responses to CIH, it does not provide direct evidence that neonatal exposure has lasting effects on heart signaling or function further into adolescence or adulthood.

## AUTHOR CONTRIBUTIONS

ECC, DM, MWK & KJS designed experiments. ECC performed treadmill stress tests and plethysmography, KJS and EC performed echocardiography. AN, IV, KK & KJS performed transcriptomics and data analysis. ECC, AN, DM, MWK, & KJS guided data interpretation. DM, MWK, & KJS secured funding. ECC, DM, MWK & KJS drafted the manuscript.

## FUNDING INFORMATION

This work was supported by UL1TR001876 from the NIH National Center for Advancing Translational Sciences and the American Heart Association 18CDA34080353 to KJS, NIGMS IDeA Networks of Biomedical Research Excellence P20GM103466 to KK, NIH R01 HL144157 to MWK, and NIH R01s HL147279, HL146169, and HL133862 to MWK and DM. Its contents are solely the responsibility of the authors and do not necessarily represent the official views of the National Center for Advancing Translational Sciences or the National Institutes of Health.

## CONFLICT OF INTEREST STATEMENT

The authors declare that they have no conflict of interest.

## ETHICS STATEMENT

Animal procedures were completed in agreement with the George Washington University institutional guidelines and in compliance with the panel of Euthanasia of the American Veterinary Medical Association and the National Institutes of Health *Guide for the Care and Use of Laboratory Animals*.

## Supporting information


Data S1.

Figure S1.

Table S1.

## Data Availability

The authors declare that the data supporting the findings of this study are available within the paper and its Supplementary Information files. Any remaining data that support the results of the study will be available from the corresponding authors upon reasonable request. Source data are provided within this paper.
