# Supplementary material for: Sex differences in cardiac transcriptomic response to neonatal sleep apnea
Source: Physiol Rep. 2024 Jul 9;12(13):e16110. doi: 10.14814/phy2.16110 (PMC11233197; doi:10.14814/phy2.16110)
Supplement: Supplementary file 2 — Figure S1. [file PHY2-12-e16110-s001.pdf]

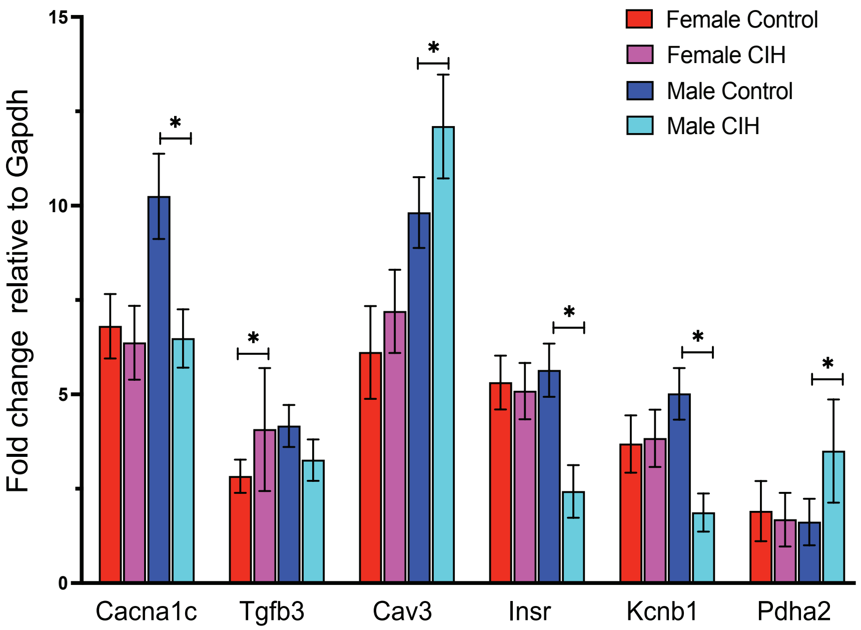

Supplemental Figure S1: Semi-quantitative rt-PCR of selected differentially expressed genes (DEGs) confirm the microarray expression fold changes.
